# Synthesis and Identification of Pentathiepin-Based Inhibitors of *Sporothrix brasiliensis*

**DOI:** 10.3390/antibiotics8040249

**Published:** 2019-12-03

**Authors:** Christopher R. M. Asquith, Ana C. S. Machado, Luisa H. M. de Miranda, Lidia S. Konstantinova, Rodrigo Almeida-Paes, Oleg A. Rakitin, Sandro A. Pereira

**Affiliations:** 1 Department of Pharmacology, School of Medicine, University of North Carolina at Chapel Hill, Chapel Hill, NC 27599, USA; 2 Laboratory of Clinical Research in Dermatozoonoses on Domestic Animals (LAPCLIN-DERMZOO), Evandro Chagas National Institute of Infectious Diseases (INI), Oswaldo Cruz Foundation (Fiocruz), Rio de Janeiro 21040-360, Brazil; ana.machado@ini.fiocruz.br (A.C.S.M.); luisa.helena@ini.fiocruz.br (L.H.M.d.M.); sandro.pereira@ini.fiocruz.br (S.A.P.); 3 Zelinsky Institute of Organic Chemistry, Russian Academy of Sciences, Moscow 119991, Russia; konstantinova_ls@mail.ru (L.S.K.); orakitin@ioc.ac.ru (O.A.R.); 4 Nanotechnology Education and Research Center, South Ural State University, Lenina Ave. 76, Chelyabinsk 454080, Russia; 5 Mycology Laboratory, INI, Fiocruz, Rio de Janeiro 21040-360, Brazil; rodrigo.paes@ini.fiocruz.br

**Keywords:** sporotrichosis, 1,2,3,4,5-pentathiepin, varacin, anti-fungal, sulfur heterocycle

## Abstract

*Sporothrix brasiliensis* is the causative agent of zoonotic sporotrichosis in Brazil and is currently referred to as the most virulent species among those of clinical importance within the genus. Sporotrichosis is an emergent disease that has come to the forefront over two decades with a recent hot spot of sporotrichosis infection emerging in the state of Rio de Janeiro. The source of these infections is now at epidemic proportions with more than 4000 cases reported in Rio de Janeiro, Brazil, alone since 1998. We developed a focused library of a rare pentathiepin ring system and identified a potent substitution pattern that yielded compounds **21** and **22**. These compounds were more potent than itraconazole which is the current standard of care for sporotrichosis.

## 1. Introduction

Sporotrichosis is an emergent disease that has come to the forefront over two decades after being first described in 1898 [1,2]. A recent hot spot of *Sporothrix* infection has emerged in the state of Rio de Janeiro, Brazil [3]. The source of these infections is now at hyperendemic proportions with more than 4000 cases reported in this region since 1998 [3,4,5]. 

*Sporothrix* species are usually non-pathogenic environmental fungi that are closely related to decaying wood, plants, and soil [6]. The majority of human and animal infections occur when the epidermis is damaged, allowing plant matter/soil, along with the fungus, to enter into the body [7]. The incidence of zoonotic infection is on the rise with feline transmission of *Sporothrix* which has been identified as the likely source of the spike in cases within Rio de Janeiro [3,4,5,8]. *Sporothrix brasiliensis* has been demonstrated to be a highly successful mammal pathogen, and it is related to zoonotic transmission from infected cats in Brazil [9]. 

Feline sporotrichosis has a varied range of clinical presentation, but it is frequently a severe condition with the development of disseminated skin lesions and respiratory involvement in infected cats [10]. Although human sporotrichosis is not usually severe, disseminated and life-threatening cases have been described in association to *S. brasiliensis* infection, especially in immunocompromised patients [11]. 

Infected cats are the most important source of *Sporothrix* transmission in the Brazilian scenario due to the high burden of yeasts in their lesions. Zoonotic transmission usually occurs through their scratches and bites [3,10]. Although the treatment of feline sporotrichosis is shown to efficiently reduce the fungal burden in lesions of cats contributing to the control of zoonotic transmission, it remains a challenge for veterinary practitioners [12]. Itraconazole frequently in association to potassium iodide is still the treatment of choice, but therapeutic failure, the occurrence of adverse effects, and recrudescence of lesions are described, raising the need for alternative therapeutic options [10].

**Figure 1:**
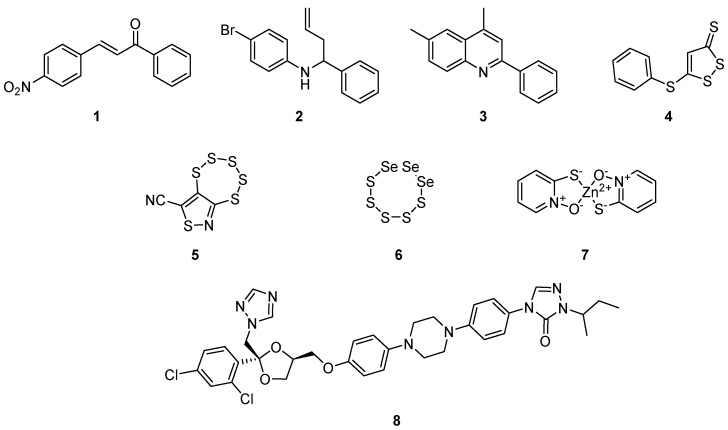
Previously reported anti-fungal compounds.

Several small anti-fungal molecules (**1**–**8**) have been reported (Figure 1), targeting different routes to treat fungal infections. The fungal cells, unlike mammalian cells, are encased in a carbohydrate-containing cell wall which has been used as a target to reduce mammalian toxicity and to target specifically the fungus [13]. The fungal cell wall has been targeted with a series of chalcone and quinoline derivatives (**1**–**3**) with varying degrees of success [14,15,16]. 

As part of those screening efforts, a series of 5-(phenylthio)-3*H*-1,2-dithiole-3-thione derivatives (**4**) was also identified, where additional sulfur substitutions improved potency [17]. This was supported by a previous report by DuPont in 1977 [18]. In this program, 7-cyano-7*H*-(1,2,3,4,5) pentathiepino(6,7-*c*)pyrazole (**5**) was developed as a potent anti-fungal in multiple crop species, providing near total control of all fungal growth [18,19,20,21,22,23]. Two of the three main compounds commercially used to suppress fungal growth are the polysulfide-containing compounds selenium sulfide (**6**) and pyrithione zinc (**7**), both used for topical applications (used to treat skin and crops respectively) [24,25,26,27].

A major interest of our work has been the reactivity of internal disulfide bridge compounds and their medicinal chemistry applications [28,29,30,31,32,33,34,35,36,37,38]. The high-density polysulfide heterocycles lend themselves to this type of redox-type applications including zinc ejection (in multiple systems) [29,30,31,32,33,34,35,36,37], anti-viral [29,30,31,32,33,34,35], cancer [36,37], and oxidative stress [38]. 

**Figure 2:**
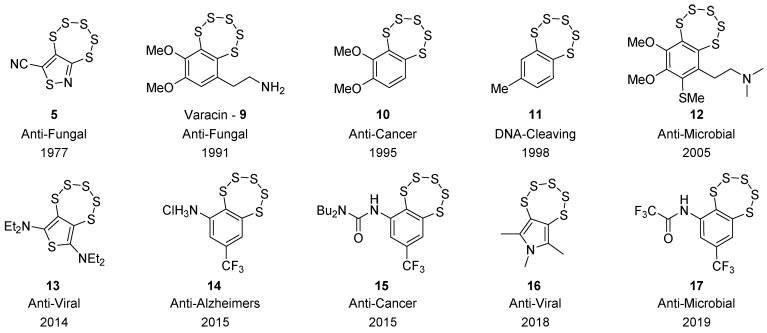
Previously reported biologically active pentathiepin compounds.

The pentathiepin functionality (**5**, **9**–**17**) has been utilized for several different indications including neoplastic diseases, Alzheimer’s, viruses, cancer, bacteria, mycoses, and as an inhibitor of protein kinase C (Figure 2) [18,19,20,21,22,23,32,39,40,41,42,43,44,45,46,47,48,49,50]. The DuPont compound **5** showed promising activity against fungus in plants with broad spectrum activity against a wider range of plant disease [18]. While the natural product varacin (**9**) showed potent inhibition of *Candida albicans* at a rate 100× that of 5-fluorouracil [39]. More recently, compound **17** has shown good inhibition activity against *S. aureus*, *C. albicans*, and *C. neoformans* with an impressive toxicity profile [50].

## 2. Results

### 2.1. Initial Investigation

To investigate pentathiepin as a viable treatment for sporotrichosis, we first screened a symmetric thiophene derivative (**13**) against six isolates of *S. brasiliensis* from skin lesions of infected cats. The antifungal susceptibility testing was based on the reference broth microdilution method according to the M38-A2 CLSI guidelines [51] with a few modifications to improve minimal inhibitory concentration (MIC) determination for *S. brasiliensis* [52]. We screened **13** at two concentrations (4 and 8 µg/mL) to determine if there was any inhibition of fungal growth. We evaluated MIC to suppress both 100% and 50% of the growth of the strains. At the higher concentration, 100% inhibition of growth was observed in all six isolates with the isolate 8607 presenting a slightly higher sensitivity and being the only isolate to respond to this compound at the lower concentration (Table 1). 

### 2.2. Synthesis of Pentathiepin Analogs

**Scheme 1:**
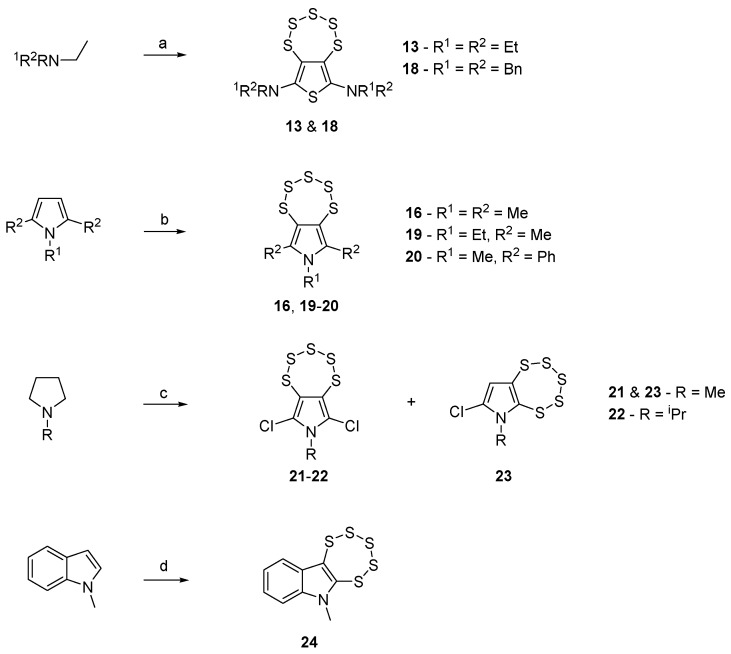
Synthesis route to pentathiepin derivatives: reagents and conditions. (**a**) 1,4-Diaza-bicyclo (2.2.2)octane (DABCO), S_2_Cl_2_, CHCl_3_, 0 °C to rt; 48 h, reflux 2 h (**13,** 30% and **18,** 3%); (**b**) DABCO, S_2_Cl_2_, CHCl_3_, 0 °C; 48 h (**16**, 36%; **19**, 40% and **20**, 62%); (**c**) DABCO, S_2_Cl_2_, CHCl_3_, 20 °C; 48 h (**21**,18%; **23**, 38% and **22**,16%); (**d**) S_2_Cl_2_ (0.8 eq), CHCl_3_, 0 °C; 48 h (**24**, 70%).

We decided to prepare a small array of pentathiepins (**13**, **16**, and **18**–**24**) (Scheme 1). The older methods for preparation of this advanced heterocycle included using an activated sulfur source, such as disulfur dichloride (S_2_Cl_2_) or trisulfur dichloride (S_3_Cl_2_) or even directly with elemental sulfur (S_8_), and adding these to an ortho-dithiol, usually under harsh conditions [53,54,55,56,57]. However, a C–H activated route utilizing the 1,4-diaza-bicyclo(2.2.2)octane (DABCO) sulfur monochloride complex enables formation of pentathiepins in one step from commercially available reagents (Scheme 1) [58,59]. The two thiophene analogs (**13** and **18**) were furnished by treating Hünig’s base and *N*,*N*-dibenzylethanamine, respectively, with DABCO and S_2_Cl_2_ for 48 h followed by refluxing with triethylamine for a further 2 h to afford **13** and **18** in good and acceptable yields, respectively [32,60]. The pyrrole derivatives were explored using a series of substituted pyrrole ring systems and treating with DABCO and S_2_Cl_2_ for 48 hours to access **16**, **19**, and **20** in good yield [61]. Treatment of *N*-methylpyrrolidine under analogous conditions allowed access to **21** and **23** in a one-pot reaction. However, under the same conditions, *N*-*iso*propylpyrrolidine produced exclusively **22** with the corresponding asymmetric product not observed [62,63,64]. This was followed by a final analog (**24**) synthesized by treating *N*-methylindole with the DABCO sulfur monochloride complex at zero degrees to furnish the final product in excellent yield (70%) [64,65].

### 2.3. Evaluation of Pentathiepin Analogs

We then screened this series of symmetrical derivatives (**16**, **18**–**22**) against eight skin lesion isolates of *S. brasiliensis* (Table 2). We found that increasing the size of the substituent on the amine of the thiophene (**18**) removed all activity. The 1,2,5-trimethyl-pyrrole (**16**) was also inactive, with an *N*-ethyl derivative (**19**) and 2,5-diphenyl derivative (**20**) also showing no activity. Interestingly, the 2,5-dichloro, *N*-methyl compound (**21**) showed some activity on two isolates with the 2,5-dichloro, *N*-isopropyl (**22**) extending this to three isolates. 

Encouraged by our early results, we screened the same set of isolates with two unsymmetrical pentathiepin derivatives (**23** and **24**) (Table 3). We found antifungal activity across all isolates with both **23** and **24**. 

**Figure 3:**
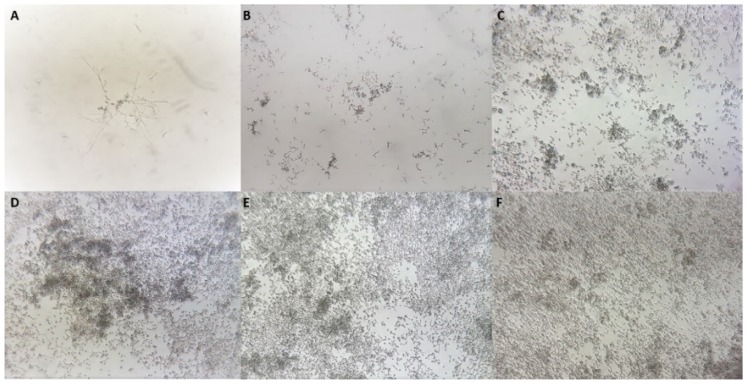
Growth of *Sporothrix brasiliensis* isolate 8584 treated with compound **24** at (**A**) 0.5 µg/mL (0.172 μM); (**B**) 0.25 µg/mL (0.086 μM); (**C**) 0.12 µg/mL (0.041 μM); (**D**) 0.06 µg/mL (0.020 μM); (**E**) 0.03 µg/mL (0.010 μM); (**F**) 0.015 µg/mL (0.005 μM).

Compound **23** presented a lower MIC in comparison to itraconazole (**8**) in 4/8 cases with the others matching or half as effective. Compound **24** was slightly less active, matching 3/8 and 1 or 2 fold less effective in the other 5/8 isolates. Compound **24** demonstrated a robust dose-dependent inhibition of *S. brasiliensis* growth. This can be observed in Figure 3 with decreasing concentrations (0.5 to 0.015 µg/mL) of **24** having a significant impact on growth of *Sporothrix brasiliensis*. 

## 3. Discussion

Sporotrichosis is still a neglected disease. It has reached a significant number of cases in Rio de Janeiro region due to the fact of its uncontrolled zoonotic spread from infected cats. However, not only has the expansion of the disease been identified in other regions in Brazil and Argentina, worldwide emergence is clear, whether related or not to zoonotic transmission. Outbreaks have been described on different continents and infected immunosuppressed patients are at serious risk. In addition, *Sporothrix brasiliensis* is the causative agent of zoonotic sporotrichosis in Brazil and is currently referred to as the most virulent species among those of clinical importance within the genus.

The sub-micro molar potencies of the pentathiepins coupled with previous reports of moderate toxicities suggests a low-risk path towards the development of a candidate compound for targeting sporotrichosis in cats and potentially humans [32]. The two asymmetric pentathiepins (**23** and **24**) both show improvements over the current standard of care (itraconazole). While the symmetrical analogs showed only weak activity, increasing the electron withdrawing nature of the substituents (methyl (**16**) versus chloro (**21**)) did increase anti-fungal potency. There is increasing evidence that the pentathiepin functionality itself is not a toxic motif and that rather the electronic contributions pendant arms are what drives non-specific toxicity. The pentathiepin functionality and other high-density sulfur heterocycles provide an exciting opportunity for the development of new clinically relevant compounds highlighted by **23** and **24**. The pentathiepin functionality, while still under-explored, has the potential to generate a pre-clinical candidate for treating sporotrichosis in vivo.

## 4. Materials and Methods

### 4.1. Isolate Collection

The isolates were previously collected from cats with sporotrichosis seen at the Laboratory of Clinical Research on Dermatozoonosis in Domestic Animals (Lapclin-Dermzoo), Evandro Chagas National Institute of Infectious Diseases (INI), Oswaldo Cruz Foundation (Fiocruz), Rio de Janeiro, Brazil. The strains were initially recovered from the exudate of ulcerated lesions or secretions from nasal cavities that were collected using a sterile swab and cultured on Sabouraud dextrose agar and Mycobiotic Agar (Difco), incubated at 25 °C and observed over 4 weeks for fungal growth. Suspected isolates were sub-cultivated on brain heart infusion agar medium (Difco) at 37 °C, and dimorphism was demonstrated by conversion to the yeast-like form. Then, the isolates were stored in sterile distilled water in the Mycology Laboratory of INI until used. 

### 4.2. General Procedures for Screening

The compounds (**13**, **16**, **18**–**24**) were tested using the broth microdilution technique in accordance with the respective reference protocols of the CSLI (Clinical and Laboratory Standards Institute). The reading of the plates was performed in wells with 100% inhibition of fungal growth (all isolates). All isolates were previously identified by the T3B-fingerprint technique as *S. brasiliensis*, and their antifungal susceptibilities to itraconazole are known [66]. Repetitions of the test were performed to confirm each result.

### 4.3. Chemistry

#### 4.3.1. General Procedure to Afford Compounds **13** and **18**

Disulfur dichloride (100 mmol) was added dropwise at −15 °C to −20 °C to a stirred solution of DABCO (100 mmol) in chloroform (40 mL). The mixture was stirred at 0 °C for 48 h. The corresponding amine (200 mmol) was added, and the mixture was refluxed for 2 h, filtered, and the solvents were evaporated. The residue was separated by column chromatography (light petroleum and then light petroleum–CH_2_Cl_2_ mixtures) to afford products **13** and **18**.

##### 6,8-*Bis*(diethylamino)thieno(3,4-*f*)(1,2,3,4,5)pentathiepin (**13**)

Orange oil (1.42 g, 30%). Anal. Calcd for C_12_H_20_N_2_S_6_ (%): C, 37.5; H, 5.2; N, 7.3; S, 50.0 Found (%) 37.6; H, 5.3; N, 7.3; S, 50.1. ^1^H NMR (300 MHz, CDCl_3_) δ: 3.17 (q, 8H, J = 7.2, 4 × CH_2_), 1.11 (t, 12H, J = 7.2, 4 × CH_3_); ^13^C NMR (75.5 MHz, CDCl_3_) 152.9 and 125.1 (2 × sp^2^ tertiary C), 50.6 (CH_2_), 13.0 (CH_3_). IR, (KBr) ν/cm^−1^: 2970 (CH), 1500, 1440, 1380, 1240, 1180, 1130, 1090, 840; *m*/*z* (EI) *m*/*z* 384 (M^+^, 46%), 320 (M^+^ −S_2_, 100%), 287 (65), 259 (95), 244 (65), 231 (69). C_12_H_20_N_2_S_6_ requires (M^+^) 383.9951, found (M^+^) 383.9943. Consistent with a previous report [60].

##### 6,8-*Bis*(dibenzylamino)thieno(3,4-*f*)(1,2,3,4,5)pentathiepin (**18**) 

Orange oil (1.68 g, 21%). Anal. Calcd for C_32_H_28_N_2_S_6_ (%): C, 60.7; H, 4.5; N, 4.4 Found (%) C, 60.4; H, 4.2; N, 4.8. ^1^H NMR (300 MHz, CDCl_3_) δ: 7.30 (s, 5H, Ph), 4.22 (s, 2H, CH_2_Ph); ^13^C NMR (75.5 MHz, CDCl_3_) 154.4, 136.9 and 127.1 (3 × sp^2^ tertiary C), 128.6, 128.4 and 127.5 (3 × CH Ph), 59.9 (CH_2_Ph). IR, (KBr) ν/cm^−1^: 3080, 3060, 3030, and 2920 (CH), 1600, 1460, 1360, 1180, 1030, 910, 740; *m*/*z* (EI) 275 632 (M+, 6%), 568 (M^+^ −S_2_, 4%), 477 (28), 356 (52), 287 (25), 91 (100). C_32_H_28_N_2_S_6_ requires (M^+^) 632.0577, found (M^+^) 632.0557. Consistent with a previous report [60].

#### 4.3.2. General Procedure to Afford Compounds **16** and **19**–**22**

Disulfur dichloride (12.5 mmol) was added dropwise at −25 °C to −35 °C to a stirred solution of DABCO (12.5 mmol) in chloroform (40 mL) under argon. The mixture was stirred at rt for 1 h. The corresponding substituted 2,5-dimethylpyrrole (5.0 mmol) in chloroform (10 mL) was added, and the mixture was stirred at 0 °C for 48 h under argon, filtered, and the solvents evaporated. The residue was separated by column chromatography (light petroleum and then light petroleum—CH_2_Cl_2_ mixtures) to afford products **16** and **19**–**22**.

6,7,8-Trimethyl-7*H*-(1,2,3,4,5)pentathiepino(6,7-*c*)pyrrole (**16**) yellow solid (4.80 g, 36%) m.p. 157–159 °C. Anal. Calcd for C_7_H_9_NS_5_ (%):C, 31.47; H, 3.40; N, 5.25. Found (%) C, 31.75; H, 3.43; N, 5.18. ^1^H NMR (300 MHz, CDCl_3_) δ: 3.40 (3H, s, CH_3_), 2.48 (6H, s, 2 × CH_3_). ^13^C NMR (75.5 MHz, CDCl_3_) 135.2 (2 × sp^2^ tertiary C), 120.5, 31.8 (CH_3_), 11.3 (2 × CH_3_). IR, (KBr) ν/cm^−1^: 2930 (C–H), 2860 (C–H), 1520, 1430, 1420, 1400, 1370, 1350, 1030, 100, 810, 780. *m*/*z* (EI) 267 (M^+^, 25), 203 (100), 170 (60), 64 (26), 56 (64). C_7_H_9_NS_5_ requires (M^+^) 266.9339, found (M^+^) 266.9331. Consistent with a previous report [61].

7-Ethyl-6,8-dimethyl-7*H*-(1,2,3,4,5)pentathiepino(6,7-*c*)pyrrole (**19**) yellow solid (5.62 g, 40%) m.p. 159–160°C. Anal. Calcd for C_8_H_11_NS_5_ (%): C, 34.17; H, 3.95; N, 4.98 Found (%) C, 34.35; H, 3.86; N, 5.09. ^1^H NMR (300 MHz, CDCl_3_) δ: 3.80 (2H, q, J = 7.2 Hz, CH_2_); 1.28 (3H, t, J = 7.2 Hz, CH_3_). ^13^C NMR (75.5 MHz, CDCl_3_) 134.4 (2 sp^2^ tertiary C) 120.8, 40.2 (CH_2_), 15.4 (CH_3_), 11.0 (CH_3_). IR, (KBr) ν/cm^−1^: 2980 (C–H), 1520, 1470, 1440, 1400, 1380, 1350, 1080, 1010, 760. *m*/*z* (EI) 281 (M^+^, 13), 249 (2), 219 (14), 217 (100), 185 (13), 184 (53), 152 (12). C_8_H_11_NS_5_ requires (M^+^) 280.9495, found (M^+^) 280.9485. Consistent with a previous report [61].

7-Methyl-6,8-diphenyl-7*H*-(1,2,3,4,5)pentathiepino(6,7-*c*)pyrrole (**20**) yellow solid (12.1 g, 62%) m.p. 252–253 °C. Anal. Calcd for C_17_H_13_NS_5_ (%): C, 52.18; H, 3.35; N, 3.58. Found (%) C, 52.35; H, 3.48; N, 3.22. ^1^H NMR (300 MHz, CDCl_3_) δ: 7.46 (10H, m, ArH), 0.34 (3H, s, CH3), ^13^C NMR (75.5 MHz, CDCl_3_) 139.9 (3 × sp^2^ tertiary C), 130.3, 131.1, 128.9 (3 × C-H), 128.5, 125.7, 34.8 (CH_3_). IR, (KBr) ν/cm^−1^: 3050, 1470, 1440, 1430, 1380, 1080, 1010, 920, 810, 780, 750, 700. *m*/*z* (EI) 391 (M^+^, 20), 327 (100), 295 (52), 280 (15), 118 (53). C_17_H_13_NS_5_ requires (M^+^) 390.9651, found (M^+^) 390.9669. Consistent with a previous report [61].

6,8-Dichloro-7-methyl-7*H*-(1,2,3,4,5)pentathiepino(6,7-*c*)pyrrole (**21**) yellow solid (5.8 g, 38%) m.p. 167–170 °C. Anal. Calcd for C_5_H_3_Cl_2_NS_5_ (%): C, 19.5; H, 1.0; N, 4.5. Found (%) C, 19.8; H, 1.2; N, 4.2. ^1^H NMR (300 MHz, CDCl_3_) δ: 3.60 (s, 3H, CH_3_); ^13^C NMR (75.5 MHz, CDCl_3_) 123.8 and 121.5 (2 × sp^2^ tertiary C), 33.2 (CH_3_). IR, (KBr) ν/cm^−1^: 1460, 1420, 1340, 1100, 780. *m*/*z* (EI) 309 (M^+^ + 2, 6), 307 (M^+^, 9), 245 (39), 243 (48). C_5_H_3_Cl_2_NS_5_ requires (M^+^) 306.8246, found (M^+^) 306.8238. Consistent with a previous report [64].

6,8-Dichloro-7-isopropyl-7*H*-(1,2,3,4,5)pentathiepino(6,7-*c*)pyrrole (**22**) yellow solid (6.4 g, 38%) m.p. 136–139 °C. Anal. Calcd for C_7_H_7_Cl_2_NS_5_ (%): C, 25.0; H, 2.1; N, 4.2. Found (%) C, 24.7; H, 1.9; N, 4.5. ^1^H NMR (300 MHz, CDCl_3_) δ: 5.01 (septet, 1H, J = 6.5, CH), 1.61 (d, 6H, J = 6.5, 2 CH_3_); ^13^C NMR (75.5 MHz, CDCl_3_) 115.3 and 78.1 (2 × sp^2^ tertiary C), 52.1 (CH), 21.7 (CH_3_). IR, (KBr) ν/cm^−1^: 2980 (CH), 1480, 1400, 1290, 1180, 1140, 795 (C–Cl); *m*/*z* (EI) 339 (M^+^ + 4, 1%), 337 (M^+^ + 2, 3%), 303 (15), 301 (22), 273 (17), 271 (22), 237 (85), 195 (100). C_7_H_7_Cl_2_NS_5_ requires (M^+^) 334.8559, found (M^+^) 334.8550. Consistent with a previous report [64].

#### 4.3.3. 7-Chloro-6-methyl-6*H*-(1,2,3,4,5)pentathiepino(6,7-*b*)pyrrole (**23**)

Sulfur monochloride (1.6 mL, 20 mmol) was added dropwise at −30 °C to −35 °C to a stirred solution of *N*-methylpyrrole (0.40 g, 5.0 mmol) and DABCO (2.24 g, 20 mmol) dissolved in chloroform (50 mL). Then, the mixture was stirred for 15 min at −20 °C and at room temperature for 48 h. The solvent was removed under reduced pressure. The residue was separated by column chromatography (light petroleum, and then light petroleum–CH_2_Cl_2_ mixtures) to produce a yellow solid (0.518 g, 1.9 mmol, 38%) m.p. 68–69 °C. Anal. Calcd for C_5_H_4_ClNS_5_ (%): C, 21.93; H, 1.47; N, 5.11. Found (%) C, 22.08; H, 1.56; N, 5.23. ^1^H NMR (300 MHz, CDCl_3_) δ: 3.71 (s, 3H, CH_3_), 6.43 (s, 1H, pyr); ^13^C NMR (75.5 MHz, CDCl_3_) 33.2 (CH_3_), 113.7 (CH), 118.8, 128.2 and 132.1 (3 sp^2^ tertiary C); *m*/*z* (EI) 275 (M^+^ + 2, 13%), 273 (M^+^, 24%, 209 (M -S_2_, 100). C_5_H_4_ClNS_5_ requires (M^+^) 272.8636, found (M^+^) 272.8643. Consistent with a previous report [32].

#### 4.3.4. 6-Methyl-6*H*-(1,2,3,4,5)pentathiepino(6,7-*b*)indole (**24**)

Disulfur dichloride (1.2 mL, 20 mmol) was added dropwise at −30 to −35 °C to a stirred solution of 1-methylindole (2.55 g, 19.5 mmol) dissolved in chloroform (50 mL). Then the mixture was stirred for 15 min at −20 °C and at 0 °C for 48 h. The mixture was refluxed for 3 h, filtered, and the solvent was removed under reduced pressure. The residue was separated by column chromatography (light petroleum, and then light petroleum–CH_2_Cl_2_ mixtures) to produce a yellow solid (0.520 g, 70%) m.p. 123–125 °C. Anal. Calcd for C_9_H_7_NS_5_ (%): 37.3; H, 2.4; N, 4.8. Found (%) C, 37.5; H, 2.3; N, 4.9. ^1^H NMR (300 MHz, CDCl_3_) δ: 7.70 (m, 1H, PhH), 7.30 (m, 1H, PhH), 3.91 (s, 3H, CH_3_). ^13^C NMR (75.5 MHz, CDCl_3_) 141.8, 137.1, 129.5 and 126.0 (4 × sp^2^ tertiary C), 125.2, 122.7, 119.6 and 111.1 (4 × CH), 32.1 (CH_3_). IR, (KBr) ν/cm^−1^: 2980 (CH), 1440, 1330, 1240, 1160, 820, 780, 740 *m*/*z* (EI) 289 (M^+^, 22%), 225 (100), 192 (42). Consistent with a previous report [64].

## Figures and Tables

**Table 1:** Initial screening of compounds **13** and **8** against six feline isolates of *Sporothrix brasiliensis*.

Compound	Growth Inhibition Percent	Isolates of *S. brasiliensis*/MIC (µg/mL) ^a^					
		8547	8584	8607	8612	8775	8729
**13**	100%	8	8	8	8	8	8
	50%	> 8	> 8	4	> 8	> 8	> 8
**8**	100%	0.5	0.5	1	1	2	2

^a^ Mean average (*n* = 4).

**Table 2:** Screening results of a symmetrical pentathiepin array against isolates of *Sporothrix brasiliensis.*

Cmpd	R	X	Isolates of *S. brasiliensis*/MIC (µg/mL) ^a^							
			8547	8726	8440	8607	8639	8775	8729	8902
**13**	N-(Et)_2_	S	8	nt	nt	4	nt	8	8	nt
**18**	N-(Bn)_2_	S	> 8	> 8	> 8	> 8	> 8	> 8	> 8	> 8
**16**	CH_3_	N–CH_3_	> 8	> 8	> 8	> 8	> 8	> 8	> 8	> 8
**19**	CH_3_	N–CH_2_CH_3_	> 8	> 8	> 8	> 8	> 8	> 8	> 8	> 8
**20**	Ph	N–CH_3_	> 8	> 8	> 8	> 8	> 8	> 8	> 8	> 8
**21**	Cl	N–CH_3_	8	> 8	> 8	> 8	8	> 8	> 8	> 8
**22**	Cl	N–C(CH_3_)_2_	8	> 8	> 8	> 8	8	> 8	> 8	8

^a^ Mean average (*n* = 4); nt = not tested.

**Table 3:** Results of asymmetric pentathiepins against isolates of *Sporothrix brasiliensis.*

Name	R	X	Isolates of *S. brasiliensis*/MIC (µg/mL) ^a^							
			8547	8726	8440	8607	8639	8775	8729	8902
**23**	Cl	N–CH_3_	1	1	1	1	0.5	0.5	1	1
**24**	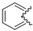	N–CH_3_	2	4	4	2	2	2	2	2
Itraconazole (**8**)			0.5	0.5	0.5	1	1	2	2	2

^a^ Mean average (*n* = 4).
